# Risk factors for oral and pharyngeal cancer in women: a study from Italy and Switzerland

**DOI:** 10.1054/bjoc.1999.0900

**Published:** 1999-12-08

**Authors:** C Bosetti, E Negri, S Franceschi, E Conti, F Levi, F Tomei, C La Vecchia

**Affiliations:** Istituto di Ricerche Farmacologiche ‘Mario Negri’, Via Eritrea 62, Milan, 20157, Italy; Centro di Riferimento Oncologico, Via Pedemontana Occ.le, Aviano (Pordenone), 33081, Italy; Servizio di Epidemiologia e Oncogenesi, Istituto ‘Regina Elena’, Rome, 00100, Italy; Registre vaudois des tumeurs, Institut universitaire de médecine sociale et préventive, CHUV-Falaises 1, Lausanne, 1011, Switzerland; Istituto di Medicina del Lavoro, Università ‘La Sapienza’, Rome, 00100, Italy; Istituto di Statistica Medica e Biometria, Università degli Studi di Milano, Via Venezian 1, Milan, 20133, Italy

**Keywords:** oral cancer, alcohol drinking, smoking, diet, case–control studies, female

## Abstract

We analysed two case–control studies of women from Italy and Switzerland, including 195 cases of oral and pharyngeal cancers and 1113 controls. The multivariate odds ratio was 4.6 for heavy smokers and 2.7 for high alcohol intake. Vegetables, fruit, β-carotene and wholegrain foods were inversely, butter and retinol directly, related to risk. © 2000 Cancer Research Campaign


*British Journal of Cancer* (2000) **82**(1), 204–207
© 2000 Cancer Research Campaign
Article no. bjoc.1999.0900

Short Communication

### Risk factors for oral and pharyngeal cancer in women: a study from Italy and Switzerland

**C Bosetti[1], E Negri[1], S Franceschi[2], E Conti[3], F Levi[4], F Tomei[5] and C La Vecchia[1,6]**

[1]Istituto di Ricerche Farmacologiche 'Mario Negri', Via Eritrea 62, 20157 Milan, Italy; [2]Centro di Riferimento Oncologico, Via Pedemontana Occ.le, 33081 Aviano (Pordenone), Italy; [3]Servizio di Epidemiologia e Oncogenesi, Istituto 'Regina Elena', 00100 Rome, Italy; [4]Registre vaudois des tumeurs, Institut universitaire de médecine sociale et préventive, CHUV-Falaises 1, 1011 Lausanne, Switzerland; [5]Istituto di Medicina del Lavoro, Università 'La Sapienza', 00100 Rome, Italy; [6]Istituto di Statistica Medica e Biometria, Università degli Studi di Milano, Via Venezian 1, 20133 Milan, Italy

**Summary** We analysed two case–control studies of women from Italy and Switzerland, including 195 cases of oral and pharyngeal cancers and 1113 controls. The multivariate odds ratio was 4.6 for heavy smokers and 2.7 for high alcohol intake. Vegetables, fruit, β-carotene and wholegrain foods were inversely, butter and retinol directly, related to risk. © 2000 Cancer Research Campaign

**Keywords**: oral cancer; alcohol drinking; smoking; diet; case–control studies; female

Most published data on oral and pharyngeal cancer relate to men, and few studies have focused on women. Among these, a re-analysis of a case–control study published by Wynder et al (1957) showed higher odds ratios (OR) for tobacco smoking in women than in men (8.1 vs 4.6). Winn et al (1981, 1984), in an investigation of women from the Southern USA, found a strong association with snuff dipping and cigarette smoking, and a protective effect of a diet rich in fruit and vegetables. A case–control study conducted in four areas of the USA (Blot et al, 1988), including 352 female cases of oral cancer, showed a strong association with cigarette smoking (OR = 6.2 for heaviest smokers) and alcohol drinking (OR = 9.1 for highest level of consumption); the relative risk for alcohol was of similar magnitude for women and men, but that for tobacco was apparently higher in women (6.2 vs 2.8 in men). With reference to diet, an inverse relation was found for fruit, vegetables, vitamin C, carotene and fibres, with similar ORs for men and women (McLaughlin et al, 1988). In a US hospital-based case–control study, including 322 women with oral cancer (Muscat et al, 1996), a strong association with smoking was observed (OR = 4.6 for the highest level of cumulative tar), and the linear trend in risk was significantly higher for women than for men. In a subset of the same study (Kabat et al, 1989), an increased risk was observed also with alcohol drinking. A recent pooled analysis of three case–control studies of oral cancer (Macfarlane et al, 1995) from the USA, Italy and China confirmed the important effect of tobacco in the aetiology of the disease, and showed that the association with tobacco in non-drinkers was stronger in women than in men. In order to better quantify the role of smoking and alcohol in oral and pharyngeal cancer among women, we analysed the combined data from two case–control studies conducted in northern Italy and Switzerland.

### SUBJECTS AND METHODS

The present analysis is based on data of two multicentre case–control studies of oral and pharyngeal cancer, the first conducted between 1984 and 1993 in the provinces of Milan and Pordenone, Italy (Negri et al, 1993), and the second between 1992 and 1997 in Pordenone and Rome, Italy, and in the Swiss Canton of Vaud (Talamini et al, 1998; Franceschi et al, 1999). These included 195 case women under 75 years (median age 57 years), with incident, histologically confirmed cancers of the oral cavity and pharynx, admitted to the major hospitals in the areas under study. The controls comprised a total of 1113 women (median age 58 years) admitted for acute, non-neoplastic conditions to the same network of hospitals. Thirty-one per cent of the controls were admitted for trauma, 28% for non-traumatic orthopaedic conditions, 21% for acute surgical disorders and 20% for miscellaneous other illnesses (including skin, eye, or ear disorders).

Data were collected by trained interviewers, using structured questionnaires, including information on socio-demographic characteristics and lifestyle habits, such as smoking and alcohol consumption, a problem-oriented medical history, frequency of intake of selected food items, including major sources of β-carotene and retinol, menstrual and reproductive factors, and lifelong use of oral contraceptives (OC) and hormone replacement therapy (HRT) in menopause. Reproducibility of the questionnaires was satisfactory (D'Avanzo et al, 1996*a*). Indices of retinol and β-carotene intake were computed using an Italian food composition database (Salvini et al, 1998).

For the analysis, controls were matched to cases by age, in quinquennia, and study centre. Odds ratios (OR) of oral and pharyngeal cancer and the corresponding 95% confidence intervals (Cl) were estimated using conditional multiple logistic regression models (Breslow and Day, 1980), including terms for education, body mass index (BMI), and alcohol and tobacco consumption, in addition to the matching variables.

### RESULTS

Table 1 shows the distribution of the 195 female cases of oral and pharyngeal cancer and the 1113 controls according to age group, education and BMI. There was no relation between education and the risk of oral cancer. Cases reported lower BMI than controls

*Received 28 April 1999*
*Revised 30 June 1999*
*Accepted 5 July 1999*

*Correspondence to:* C Bosetti

**Table 1** Distribution of women with 195 oral and pharyngeal cancer cases and 1113 controls according to age, education and body mass index. Italy and Switzerland, 1984–1997

| | Cases | | Controls | | OR[a] (95% CI) |
|---|---|---|---|---|---|
| | No. | % | No. | % | |
| Age | | | | | |
| < 45 | 19 | 9.8 | 191 | 17.2 | |
| 45–54 | 57 | 29.2 | 276 | 24.8 | |
| 55–64 | 72 | 36.9 | 332 | 29.8 | |
| ≥ 65 | 47 | 24.1 | 314 | 28.2 | |
| Education (years)[c] | | | | | |
| < 7 | 122 | 62.6 | 709 | 63.9 | 1[b] |
| 7–11 | 40 | 20.5 | 265 | 23.9 | 0.79 (0.47–1.33) |
| ≥ 12 | 33 | 16.9 | 136 | 12.2 | 1.07 (0.60–1.91) |
| Body mass index (kg/m$^2$)[c] | | | | | |
| ≥ 26.30 | 54 | 27.8 | 383 | 34.7 | 1[b] |
| 22.95–26.30 | 57 | 29.4 | 373 | 33.8 | 0.99 (0.64–1.53) |
| < 22.95 | 83 | 42.8 | 348 | 31.5 | 1.50 (0.97–2.33) |

[a]Estimates from conditional logistic regression, adjusted for education, body mass index, tobacco and alcohol consumption. [b]Reference category. [c]The sum does not add up to the total because of some missing values.

**Table 2** Distribution of women with 195 oral and pharyngeal cancer cases and 1113 controls according to tobacco and alcohol consumption, and corresponding odds ratio (OR) and 95% confidence intervals (CI). Italy and Switzerland, 1984–1997

| | Cases | Controls | OR[a] (95% CI) |
|---|---|---|---|
| Tobacco smoking | | | |
| Never smokers | 72 | 768 | 1[b] |
| Ex-smokers | 19 | 111 | 1.60 (0.90–2.85) |
| Current smokers[c] | | | |
| 1–14 (cigarettes per day) | 57 | 139 | 3.63 (2.34–5.64) |
| ≥ 15 (cigarettes per day) | 47 | 93 | 4.56 (2.72–7.62) |
| $\chi^2$ trend[d] (*P*-value) | | | 42.1 (<0.0001) |
| Duration of smoking[c,d] (years) | | | |
| <28 | 29 | 138 | 1.64 (1.01–2.67) |
| ≥28 | 75 | 88 | 5.08 (3.34–7.71) |
| $\chi^2$ trend (*P*-value) | | | 55.6 (<0.0001) |
| Alcohol intake[c] (drinks per day) | | | |
| 0 | 32 | 381 | 1[b] |
| <2 | 42 | 339 | 1.45 (0.86–2.43) |
| ≥2 | 121 | 392 | 2.74 (1.71–4.38) |
| $\chi^2$ trend (*P*-value) | | | 19.7 (<0.0001) |

[a]Estimates from conditional logistic regression, adjusted for education, body mass index, tobacco and alcohol consumption. [b]Reference category. [c]The sum does not add up to the total because of some missing values. [d]Omitting ex-smokers.

(OR = 1.50 for women in the lowest tertile of BMI compared to the highest one). Compared to never smokers, the OR was 3.63 for moderate smokers (< 15 cigarettes per day), 4.56 for heavy smokers (≥ 15 cigarettes per day), and 1.60 for ex-smokers. Among current smokers, there was a significant trend in risk with dose. With reference to the duration of smoking, the OR was 1.64 for women who had been smoking for fewer than 28 years, and 5.08 for 28 years or longer, again with a significant trend in risk. Compared to non-alcohol drinkers, the ORs were 1.45 for women drinking < 2 drinks per day and 2.74 for ≥ 2 drinks per day (Table 2).

The risk of oral cancer increased in subsequent levels of smoking with alcohol intake, and was also related to smoking across each level of alcohol consumption. The OR was 3.14 for non-drinking heavy smokers, and 1.83 for never smoking heavy drinkers, compared to never smokers non-drinkers; the OR for heavy smokers and heavy alcohol drinkers, compared to never smokers and never alcohol drinkers, was approximately 15 (Table 3).

Table 4 gives the distribution of cases and controls by approximate tertiles of consumption of selected foods and micronutrients. Green vegetables, fresh fruit, β-carotene and wholegrain foods were inversely related to oral cancer: the ORs for the highest tertile of intake, compared to the lowest, were 0.25 for vegetables, 0.58 for fruit, 0.54 for β-carotene and 0.63 for wholegrain foods. Butter and, though not significantly, retinol were directly related to oral cancer, with ORs of 1.89 and 1.48 respectively, for the highest consumption level.

Menstrual, reproductive and hormonal factors are presented in Table 5. No appreciable association was observed with age at menarche, menstrual cycles pattern, number of abortions and age at first pregnancy. Cases reported earlier age at menopause: compared to women with menopause before 50 years, the multivariate OR was 0.46 for those with menopause at 50 years or over. The OR was 0.58 in parous women compared to nulliparous ones. The inverse relation with age at menopause was observed in smokers (OR = 0.4), as well as in never smokers (OR = 0.5), and was somewhat stronger in women with a BMI < 25 (OR = 0.3) than in those with a BMI ≥ 25 (OR = 0.7). Likewise the ORs for parous women were below unity in smokers (OR = 0.7) and in never smokers (OR = 0.5), as well as in strata of BMI. The ORs were below unity also for OC (OR = 0.73) and HRT (OR = 0.88); none of these estimates, however, was significant.

**Table 3** Combined effect of cigarette smoking and alcohol intake on the risk of oral and pharyngeal cancer in women. Italy and Switzerland, 1984–1997

| Alcohol intake (drinks per day) | Odds ratio[a] for number of cigarettes per day [No. cases:No. controls] | | |
|---|---|---|---|
| | Never smoker | 1–14 | ≥15 |
| 0 | 1[b] [16:272] | 2.25 [5:35] | 3.14 [7:39] |
| < 2 | 1.92 [27:235] | 2.54 [6:44] | 2.04 [4:22] |
| ≥ 2 | 1.83 [29:260] | 11.55 [46:60] | 15.41 [36:32] |

[a]Estimates from conditional logistic regression, adjusted for education and body mass index. [b]Reference category.

*© 2000 Cancer Research Campaign*

**Table 4** Distribution of women with 195 oral and pharyngeal cancer cases and 1113 controls according to approximate tertiles of consumption of selected foods and micronutrients, and corresponding odds ratio (OR) and 95% confidence intervals (CI). Italy and Switzerland, 1984–1997

| | Frequency of consumption No. of cases:No. of controls | | | OR[a,b] (95% CI) | | $\chi^2$ trend (*P*-value) |
|---|---|---|---|---|---|---|
| | 1 (low) | 2 (moderate) | 3 (high) | 2 | 3 | |
| Green vegetables[c] | 47:199 | 70:469 | 48:407 | 0.59 (0.37–0.96) | 0.25 (0.15–0.44) | 25.5 <0.0001 |
| Fresh fruit | 57:176 | 50:297 | 88:640 | 0.66 (0.40–1.09) | 0.58 (0.37–0.89) | 5.7 0.02 |
| β-carotene | 95:327 | 46:380 | 54:406 | 0.55 (0.35–0.85) | 0.54 (0.34–0.86) | 6.8 <0.01 |
| Retinol | 44:388 | 68:364 | 83:361 | 1.50 (0.95–2.35) | 1.48 (0.95–2.31) | 2.8 0.10 |
| Wholegrain foods[c,d] | 69:457 | 10:134 | – | 0.63 (0.30–1.32) | – | |
| Butter[c,d] | 39:393 | 40:198 | – | 1.89 (1.11–3.20) | – | |

[a]Estimates from conditional logistic regression, adjusted for education, body mass index, tobacco and alcohol consumption. [b]First (low) tertile of consumption as reference category. [c]The sum does not add up to the total because of some missing values. [d]Based on 79 cases and 592 controls.

**Table 5** Distribution of women with 195 oral and pharyngeal cancer and 1113 controls according to menstrual and reproductive and hormonal factors, and corresponding odds ratio (OR) and 95% confidence intervals (CI). Italy and Switzerland, 1984–1997

| | Cases | Controls | OR[a] (95% CI) |
|---|---|---|---|
| Age at menarche[c] | | | |
| <15 | 157 | 884 | 1[b] |
| ≥15 | 38 | 224 | 0.67 (0.43–1.04) |
| Menstrual cycles[c] | | | |
| Regular | 181 | 1002 | 1[b] |
| Irregular | 14 | 102 | 0.97 (0.50–1.86) |
| Age at menopause[c,d] | | | |
| < 50 | 100 | 554 | 1[b] |
| ≥ 50 | 53 | 420 | 0.46 (0.30–0.70) |
| Number of births[c] | | | |
| 0 | 49 | 172 | 1[b] |
| ≥ 1 | 145 | 931 | 0.58 (0.37–0.92) |
| Number of abortions[c] | | | |
| 0 | 145 | 793 | 1[b] |
| ≥ 1 | 49 | 309 | 0.92 (0.62–1.37) |
| Age at first pregnancy[c] | | | |
| < 25 | 89 | 521 | 1[b] |
| ≥ 25 | 62 | 435 | 1.03 (0.69–1.56) |
| Oral contraceptives use[c] | | | |
| Never | 180 | 1002 | 1[b] |
| Ever | 15 | 107 | 0.75 (0.38–1.47) |
| Hormone replacement therapy use[c,d] | | | |
| Never | 136 | 701 | 1[b] |
| Ever | 17 | 67 | 0.88 (0.45–1.72) |

[a]Estimates from conditional logistic regression, adjusted for education, body mass index, tobacco and alcohol consumption. [b]Reference category. [c]The sum does not add up to the total because of some missing values. [d]Post-menopausal women only.

### DISCUSSION

This study, based on one of the largest available datasets on oral and pharyngeal cancer in women, confirms that tobacco smoking and high alcohol consumption are the major risk factors for the disease in women as in men in developed countries. The data also support the hypothesis that the simultaneous exposure to alcohol and tobacco has a multiplicative effect on oral carcinogenesis (Blot et al, 1988; Doll et al, 1993). Elevated consumption of both alcohol and tobacco produced an approximately 15-fold increased risk in women. Moreover, this study indicates that oral cancer is associated with alcohol drinking in never smokers and tobacco smoking in non-drinkers (Blot et al, 1988; Talamini et al, 1990; Ng et al, 1993).

However, certain quantitative differences between women and men are of potential interest. In a companion study on males (Franceschi et al, 1990), the OR for men was 5.3 for smokers <15 cigarettes per day, and 14.3 for smokers of ≥15 cigarettes per day; for alcohol consumption, the OR was 1.1 for 3–4 drinks per day, 3.2 for 5–8 drinks and 3.4 for ≥ 9 drinks per day. The apparently lower ORs for tobacco smoking in women are attributable to higher tobacco consumption in males within each category. The ORs for alcohol were somewhat higher than those of men, possibly on account of differential underreporting of alcohol consumption of males and females, since moderate alcohol drinking is less socially accepted in women than men. Also, women may be more susceptible than men to alcohol carcinogenesis (Blume, 1986).

This study confirms (Kabat et al, 1994; D'Avanzo et al, 1996*b*) that women with oral cancer tend to have a lower BMI, though this could be partly a consequence, rather than a cause, of the oral lesions. Our investigation also confirms that green vegetables, fresh fruit, β-carotene (Zheng et al, 1993), and wholegrain foods (McLaughlin et al, 1988) have a favourable, and that butter and retinol (McLaughlin et al, 1988; Marshall et al, 1992) have an unfavourable effect on oral carcinogenesis in women. Since the inverse association of β-carotene and oral cancer risk was weaker than that of vegetables, it is possible that this association was not specific, but reflected a generally poorer nutritional status in the cases. The inverse association with wholegrain foods can be explained by their high contents of several micronutrients and fibres with potential favourable effects on cancer risk (Chatenoud et al, 1998).

The direct association with butter was also found in a study of both males and females which included part of this dataset

 © 2000 Cancer Research Campaign

(Franceschi et al, 1999), as well as in a multicentre investigation of laryngeal and hypopharyngeal cancer (Estève et al, 1996); other studies have reported an increased risk of oral cancer in women associated with total fats and saturated fats (McLaughlin et al, 1988; Marshall et al, 1992).

Scanty information is available on the potential role of reproductive and hormonal factors on oral cancer risk. In this study, the only such factors significantly associated with oral cancer risk were parity and age at menopause, (Baron et al, 1990) which are inversely related to tobacco smoking. The risk estimates for exogenous hormones (OC and HRT) were based on very few exposed women, but, as for gastric (La Vecchia et al, 1994) and colorectal cancer (Fernandez et al, 1998*a*, 1998*b*), the findings are reassuring.

A possible limitation of this study is the use of hospital controls, since smokers may be admitted to hospital more frequently than non-smokers (La Vecchia et al, 1988). This potential bias, however, would lead to an underestimate of the strength of the real association. The use of hospital controls should reduce recall bias, and increase the comparability of information obtained by cases and controls (D'Avanzo et al, 1996*a*). Among the strengths of this study are the practically complete participation rate, the similar catchment areas of cases and controls, and the large dataset in Western countries.

In conclusion, the results of this study are in broad agreement with available data, mainly from the USA (Winn et al, 1984; Blot et al, 1988; McLaughlin et al, 1988), indicating that, despite the much lower incidence in women than in men, the major risk and protective factors for oral and pharyngeal cancer are similar for both sexes, with a predominant role of tobacco and alcohol. In terms of population attributable risk (Bruzzi et al, 1985) tobacco smoking accounted for 44% of all oral cancers, alcohol drinking for 46%, and the combination of the two factors for 69%. With regard to diet, the estimated population attributable risks were 46% for low vegetable, 16% for low fruit, 22% for low β-carotene consumption and 24% for high butter intake.

### ACKNOWLEDGEMENTS

This work was conducted with the contribution of the Italian League Against Cancer, the Italian Association for Cancer Research, Milan, the Swiss Foundation for Research Against Cancer (Contract Grants AKT 413 and 700), and the Vaud League Against Cancer. The authors thank Mrs P Bonifacino and Mrs J Baggott for editorial assistance.

### REFERENCES

Baron JA, La Vecchia C and Levi F (1990) The antiestrogenic effect of cigarette smoking in women. *Am J Obstet Gynecol* **162**: 502–514

Blot WJ, McLaughlin JK, Winn DM, Austin DF, Greenberg RS, Preston-Martin S, Bernstein L, Schoenberg JB, Stemhagen A and Fraumeni JF Jr (1988) Smoking and drinking in relation to oral and pharyngeal cancer. *Cancer Res* **48**: 3282–3287

Blume SB (1986) Women and alcohol: a review. *JAMA* **256**: 1467–1470

Breslow NE and Day NE (1980) *Statistical Methods in Cancer Research, Vol. 1. The Analysis of Case–control Studies*. IARC Scientific Publication 32. IARC: Lyon, France

Bruzzi P, Green SB, Byar DP, Brinton LA and Schairer C (1985) Estimating the population attributable risk for multiple risk factors using case–control data. *Am J Epidemiol* **122**: 904–914

Chatenoud L, Tavani A, La Vecchia C, Jacobs DR Jr, Negri E, Levi F and Franceschi S (1998) Whole grain food intake and cancer risk. *Int J Cancer* **77**: 24–28

D'Avanzo B, La Vecchia C, Katsouyanni K, Negri E and Trichopoulos D (1996*a*) Reliability of information on cigarette smoking and beverage consumption provided by hospital controls. *Epidemiology* **7**: 312–315

D'Avanzo B, La Vecchia C, Talamini R and Franceschi S (1996*b*) Anthropometric measures and risk of cancers of the upper digestive and respiratory tract. *Nutrition Cancer* **26**: 219–227

Doll R, Forman D, La Vecchia C and Woutersen R (1993) Alcoholic beverages and cancers of the digestive tract and larynx. Health issues related to alcohol consumption, pp. 126–166. ILSI Press: Washington

Estève J, Riboli E, Péquignot G, Terracini B, Merletti F, Crosignani P, Ascunce N, Zubiri L, Blanchet F, Raymond L, Repetto and Tuyns AJ (1996) Diet and cancers of the larynx and hypopharynx: the IARC multi-center study in southwestern Europe. *Cancer Causes Control* **7**: 240–252

Fernandez E, La Vecchia C, Franceschi S, Braga C, Talamini R, Negri E and Parazzini F (1998*a*) Oral contraceptive use and risk of colorectal cancer. *Epidemiology* **9**: 295–300

Fernandez E, La Vecchia C, Franceschi S, Braga C, Talamini R, Negri E and Parazzini F (1998*b*) Hormone replacement therapy and risk of colon and rectal cancer. *Cancer Epidemiol Biomarkers Prev* **7**: 329–333

Franceschi S, Talamini R, Barra S, Baron AE, Negri E, Bidoli E, Serraino D and La Vecchia C (1990) Smoking and drinking in relation to cancers of the oral cavity, pharynx, larynx and esophagus in Northern Italy. *Cancer Res* **50**: 6502–6507

Franceschi S, Favero A, Conti E, Talamini R, Volpe R, Negri E, Barzan L and La Vecchia C (1999) Food groups, olive oil and other fats and cancer of the oral cavity and pharynx. *Br J Cancer* **80**: 614–620

Kabat GC, Herbert JR and Wynder EL (1989) Risk factors for oral cancer in women. *Cancer Res* **49**: 2803–2806

Kabat GC, Chang CJ and Wynder EL (1994) The role of tobacco, alcohol use, and body mass index in oral and pharyngeal cancer. *Int J Epidemiol* **23**: 1137–1144

La Vecchia C, Pagano R, Negri E and Decarli A (1988) Smoking and prevalence of disease in the 1983 Italian National Health Survey. *Int J Epidemiol* **17**: 50–55

La Vecchia C, D'Avanzo B, Franceschi S, Negri E, Parazzini F and Decarli A (1994) Menstrual and reproductive factors and gastric cancer risk in women. *Int J Cancer* **59**: 761–764

Macfarlane GJ, Zheng T, Marshall JR, Boffetta P, Niu S, Brasure J, Merletti F and Boyle P (1995) Alcohol, tobacco, diet and the risk of oral cancer: a pooled analysis of three case-control studies. *Eur J Cancer* **31B**: 181–187

McLaughlin JK, Gridley G, Block G, Winn DM, Preston-Martin S, Schoenberg JB, Greenberg RS, Stemhagen A, Austin DF, Ershow AG, Blot WJ and Fraumeni JF Jr (1988) Dietary factors in oral and pharyngeal cancer. *J Natl Cancer Inst* **80**: 1237–1243

Marshall JR, Graham S, Haughey BP, Shedd D, O'Shea R, Brasure J, Wilkinson GS and West D (1992) Smoking, alcohol, dentition and diet in the epidemiology of oral cancer. *Eur J Cancer, Part B, Oral Oncol* **28B**: 9–15

Muscat JE, Richie JP Jr, Thompson S and Wynder E (1996) Gender differences in smoking and risk of oral cancer. *Cancer Res* **56**: 5192–5197

Negri E, La Vecchia C, Franceschi S and Tavani A (1993) Attributable risk for oral cancer in northern Italy. *Cancer Epidemiol Biomarkers Prev* **2**: 189–193

Ng SK, Kabat GC and Wynder EL (1993) Oral cavity cancer in non-users of tobacco. *J Natl Cancer Inst* **85**: 743–745

Salvini S, Parpinel M, Gnagnarella P, Maisonneuve P and Turrini A (1998) *Banca dati di composizione degli alimenti per studi epidemiologici in Italia*. Istituto Europeo di Oncologia: Milan, Italy

Talamini R, Franceschi S, Barra S and La Vecchia C (1990) The role of alcohol in oral and pharyngeal cancer in non-smokers and of tobacco in non-drinkers. *Int J Cancer* **46**: 391–393

Talamini R, La Vecchia C, Levi F, Conti E, Favero A and Franceschi S (1998) Cancer of the oral cavity and pharynx in nonsmokers who drink alcohol and in nondrinkers who smoke tobacco. *J Natl Cancer Inst* **90**: 1901–1903

Winn DM, Blot WJ, Shy CM, Pickle LW, Toledo A and Fraumeni JF Jr (1981) Snuff dipping and oral cancer among women in the southern United States. *N Engl J Med* **304**: 745–749

Winn DM, Ziegler RG, Pickle LW, Gridley G, Blot WJ and Hoover RN (1984) Diet in the etiology of oral and pharyngeal cancer among women from the Southern United States. *Cancer Res* **44**: 1216–1222

Wynder EL, Bross IJ and Feldman RM (1957) A study of the etiological factors in cancer of the mouth. *Cancer* **10**: 1300–1327

Zheng W, Blot WJ, Diamond EL, Norkus EP, Spate V, Morris JS and Comstock GW (1993) Serum micronutrients and the subsequent risk of oral and pharyngeal cancer. *Cancer Res* **53**: 795–798

© 2000 Cancer Research Campaign

